# Simulated larvae dispersion of the invasive sun-coral (*Tubastrea* spp.) along Rio de Janeiro’s coast: The role of submesoscale filaments on offshore transport and connectivity

**DOI:** 10.1371/journal.pone.0313240

**Published:** 2025-06-05

**Authors:** Leandro Calado, Bernardo Cosenza, Francisco Moraes, Caique Dias Luko, Damián Mizrahi, Fabio C. Xavier, Daniela Batista, Roberto Domingos, Sávio Calazans, Fernanda Araújo, Ricardo Coutinho

**Affiliations:** 1 Admiral Paulo Moreira Marine Research Institute (IEAPM), Brazilian Navy, Arraial do Cabo, 28930-000, Rio de Janeiro, Brazil; 2 Department of Oceanography, University of Rio de Janeiro State (UERJ), 20550-900, Brazil; 3 Scripps Institution of Oceanography, University of California San Diego, La Jolla, California, United States of America; 4 Department of Computational Modeling, Polytechnic Institute - University of Rio de Janeiro State (IPRJ-UERJ), Brazil; Universidade de Aveiro, PORTUGAL

## Abstract

The spread of invasive species in marine ecosystems is a growing global concern, particularly in regions with high economic and ecological importance. Sun corals (*Tubastraea* spp.) are native scleractinians from the Pacific Ocean that have spread along most of the Brazilian coast. This invasive species initially established populations in Rio de Janeiro state, SE Brazil, reaching high levels of abundance. Although the ecological aspects and impacts caused by this organism have been studied in detail, the natural mechanisms that drive its dispersal have attracted little attention. In this research, we focus on the coastal dispersion of sun coral larvae between Cabo de São Tomé and Ilha Grande Bay, and the offshore transport of sun coral larvae, investigating how submesoscale oceanographic features such as filaments, fronts and eddies influence connectivity among different sites. A high-resolution numerical model was used to simulate the coastal dynamics, incorporating the influence of the Brazil Current, wind-driven circulation, and submesoscale structures. Larval dispersal was examined under different wind scenarios, including northeasterly winds that drive southward currents and enhance offshore transport via submesoscale filaments. Results show that submesoscale features, particularly filaments emerged from upwelling regions, play a significant role on sun coral larvae dispersion. These features act as pathways that connect larvae from coastal to offshore oil exploration areas, highlighting the importance of both natural and anthropogenic processes for the dispersal of this invasive species. This research provides critical insights into the mechanisms governing the spread of invasive marine species, emphasizing the need for integrated coastal management strategies. Understanding how physical processes drive larval transport is essential for developing targeted control measures to mitigate the impact of invasive species like sun coral on native ecosystems and local economies. Furthermore, the study underscores the importance of monitoring both natural and anthropogenic influences on marine bioinvasions, particularly in regions with significant offshore industrial activities.

## Introduction

**Fig 1:**
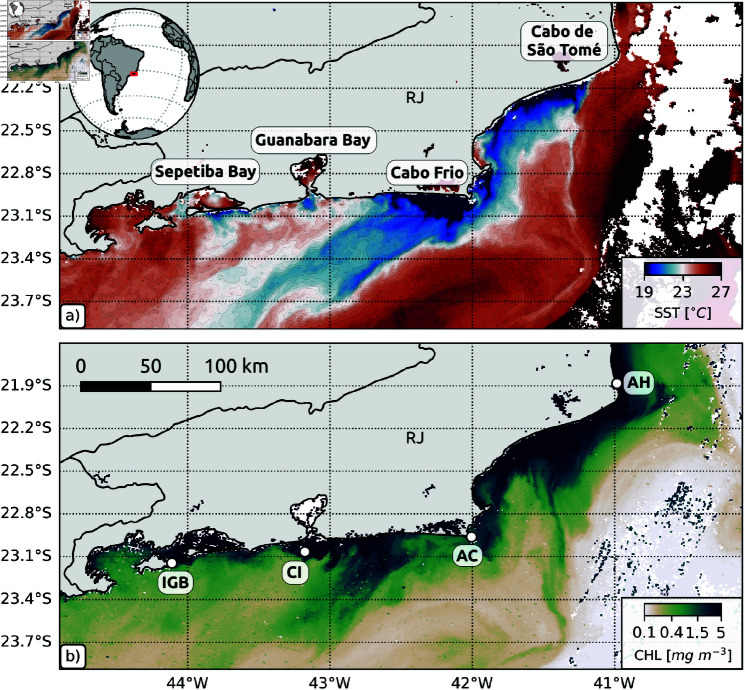
Submesoscale filaments along the coast of Rio de Janeiro : (**a**) sea surface temperature (SST) in May 07 2024; and (**b**) chlorophyll concentration (CHL) in May 08 2024. Level 2 satellite images from Sentinel 3A. This figure also indicates the locations of Açu Harbor (AH), Arraial do Cabo (AC), Cagarras Islands (CI), Ilha Grande Bay (IGB), as well as Sepetiba Bay, Guanabara Bay, Cabo Frio, and Cabo de São Tomé, all situated in the state of Rio de Janeiro (RJ). These maps are based on EUMETSAT Level 2 SLSTR and OCLI data distributed in 2024.

There is a growing concern regarding the spread of non-indigenous organisms in coastal regions. In Brazil, numerous studies assessed the impact that biological invasions have on the biodiversity of native flora and fauna, as well as on the economic activities associated with it [1,2]. The sun coral (*Tubastraea* spp.), has been a subject of increasing research over the past two decades [3], with its origins traced to the Pacific Ocean. This genus has spread initially throughout the Caribbean Sea in 1943, Gulf of Mexico in 1977 and the Southwestern Atlantic in the late 80’s, establishing a population in the Campos Basin, off Rio de Janeiro [4,5]. These corals have been reported all along Rio’s coast, in areas such as Arraial do Cabo (AC), Ilha Grande Bay (IGB), Açú Harbor (AH) and Cagarras Islands (CI), where populations have been established since the 1990s [1,5,6], see Fig 1. Once new populations are established, fertile adult colonies emit planktonic larvae - planulae - that are dispersed by ocean currents, since their swimming capacity is restricted at the microhabitat scale [3,4,6–8].

Although the coast of the state of Rio de Janeiro is densely populated by sun corals, the arrival of larvae from the open ocean is limited in this region [9]. Recently a comprehensive evaluation led by [10] focused on the distribution of sun coral larvae in the oceanic region of Cabo Frio. This study placed emphasis on their interaction with the Brazil Current (BC) and its influence by mesoscale dynamics. The findings from that study have illuminated that the BC can act as a dynamic barrier separating offshore and coastal areas. This discovery raises the hypothesis that the colonization of sun Coral on the coast may be influenced by vectors responsible for transporting the larvae to regions in close proximity to the coastal areas. This underscores the importance of understanding how coral dispersal mechanisms can be articulated or act synergistically.

The continental shelf in southeast Brazil can be categorized into outer, middle, and inner shelf, as detailed by [11]. From north of Cabo de São Tomé (CST) to Cabo Frio (CF) region, in contrast to the remainder of southeastern Brazil, there is a notable narrow continental shelf. Consequently, oceanic features like the BC with its meanders and eddies, exerts more influence on nutrient, plankton and physical properties exchanges between the continental shelf and open ocean [12]. South of Rio de Janeiro State, the continental shelf becomes wider, a pattern that extends all the way to the southernmost part of Brazil, where open ocean processes are not dominant. However, the frequency of incidence and intensity of cold fronts marked by strong southwest winds is highly frequent. As a result, a comprehensive understanding of the coastal region must account for the intricacies of the continental shelf itself, which are influenced by the wind patterns, mesoscale dynamics of the Brazil Current and submesoscale processes near Cabo Frio region.

This coastal area has experienced a different dynamic of sun coral spread, which has been slowed down compared to other locations, mainly in the Arraial do Cabo area. These circumstances are noteworthy due to their unique nature, as they appear to be stable despite their exotic origins. However, uncovering the potential primary sources of this colonization points towards connections with offshore oil and gas exploration along the southeastern coast of Brazil. This may arise from the support equipment that frequently docks in coastal ports or from the potential influx of larvae from oil and gas exploration zones.

*Tubastraea* spp. can be effectively spread through offshore oil and gas operations, particularly via vessels and industrial structures. As noted by [3], *Tubastraea* spp. have shown a strong ability to survive on stationary, slow-moving structures such as oil platforms, support vessels, and equipment, especially those used in the oil and gas offshore industry. This observation implies that support vessels can play a major role as vectors for the introduction of *Tubastraea* spp., facilitating their spread along the Brazilian coast. According to [13], there is evidence that mechanically removing sun coral colonies can lead to significant larval releases during high fecundity periods. This on-site evidence underscores the need for cautious consideration when opting for mechanical removal as the preferred method to curb the proliferation of sun coral. These techniques may lead to additional risks of anthropogenic dissemination due to the regenerative capacity of coral fragments [14,15] and the release response of large numbers of larvae as a result of the stress caused by mechanical removal [13]. This is a possible explanation for the high abundance of this invasive organism in IGB [16,17], since mechanical sun coral removal protocols have historically been practiced in the IGB nautical center that supports offshore oil and gas activity [18].

The expansion of offshore oil and gas exploration areas may facilitate the oceanic dispersion of sun coral, once it leads to an increase in the availability of consolidated substrate on the oil and gas exploration areas. [10] showed that in the BC domain some larvae interact with Cabo Frio eddies, which restrict their dispersal over the continental shelf, while others are captured by the BC, increasing their dispersal and keeping them offshore. The study on the dispersion of sun coral larvae conducted by [19] was restricted to the Arraial do Cabo area and did not address dispersal and connectivity with other already affected coastal regions. Furthermore, when it comes to coastal-offshore exports it is not clear if the coastal populations can be a larval source to colonize offshore structures.

The wind regime influences the continental shelf dynamics, determining the direction of the surface coastal currents [11]. Coastal upwelling in the Arraial do Cabo offshore region is primarily driven by persistent northeast winds, while southwest winds from cold fronts contribute to coastal subsidence, also influencing the direction of currents on the continental shelf. This change in currents due to wind regime, especially during periods of cold front entry, likely might drive the dispersion of larvae in the region. Furthermore, although the coastal region has its own dynamics, [20] and [21], show that the interaction among the eddies of the Brazil Current plays a fundamental role in coastal dynamics.

In addition to the influence of winds and BC meanders on the shelf dynamics, submesoscale phenomena also play a major role on the circulation. Submesoscale currents have typical length scales that range from 100s m-10s km [22], and are dynamically defined as currents with 𝒪(1) Rossby numbers (Ro=ζ/*f*, where ζ is relative vorticity and *f* is the planetary vorticity). Submesoscale phenomena can be triggered by flow-topography interactions, especially in separation regions where enhanced lateral shear against topography ejects filaments and eddies [23–27]. Nearshore lagrangian transport and connectivity are intimately linked to submesoscale currents, which aggregate material [28,29] and open up transport pathways across the shelf [30].

Submesoscale filaments associated with the Cabo Frio upwelling system have been reported as common features observed in numerical simulations [31]. The observation of these submesoscale filaments has been a challenge due to their small spatial scales, and to local SST biases in microwave-based satellite datasets [32]. However, in clear-sky conditions, these filaments can be seen using the high-resolution data from the Sentinel 3 satellite (available at https://data.eumetsat.int). We use the Sentinel-3A Level 2 SLSTR (Sea and Land Surface Temperature Radiometer) Near-Real Time SST product (1 km resolution at nadir), and the Level 2 OCLI (Ocean and Land Colour Instrument) Near-Real Time Chlorophyll Reduced Resolution product (1.2km resolution at nadir) to show these filaments in May 07 and 08 2024 (Fig 1a). Cold submesoscale filaments, arising from Cabo Frio ($23^{\circ }$S) and Cabo de São Tomé ($22^{\circ }$S), are associated to strong chlorophyll concentrations (Fig 1b), and might be essential for the cross-shelf export of water properties, carbon and larvae in the region. In particular, the filaments must be key features for the regional dispersal of sun coral larvae since populations have been reported in Arraial do Cabo and Açu Harbor.

In this study, we investigate the role of submesoscale phenomena in establishing transport pathways and connectivity of sun coral along the coast of Rio de Janeiro (Brazil). Our goal is to identify how the continental shelf circulation controls coastal inter-connectivity between colonized regions, and to test the offshore larvae exportation hypothesis. We focus our investigation in two realistic case studies where different wind conditions are applied. In these scenarios, different wind forcing modulates the submesoscale activity by changing the shelf circulation. We conducted experiments, building upon the numerical implementation presented by [10], with the aim of investigating the interchangeability of sun coral larvae within the coastal region. This implementation involved the incorporation of a coastal grid with an approximate resolution of 600 m. In the Methods section, we present the numerical model we implemented and provide details on the larval dispersal experiments. Next, we present and discuss the Results, and finally, we offer the Conclusions.

## Methods

### Study area and hydrodynamic model

**Fig 2:**
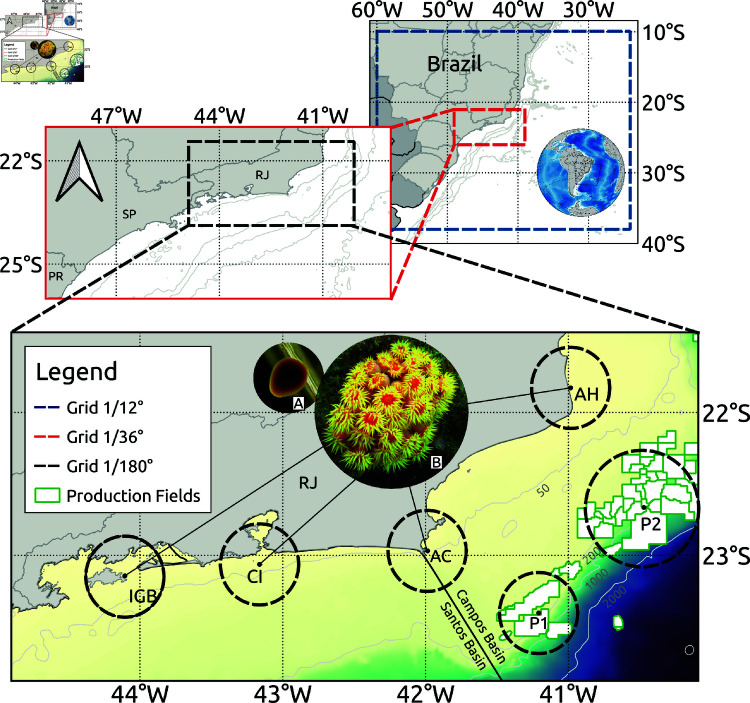
Model grids representing the study area. The coarse-resolution grids used for nesting are shown, with the higher-resolution grid highlighting the four locations of larvae release—IGB, CI, AC and AH. The offshore oil production fields are the green polygons, gray lines are the isobaths. (**A**) photo of the sun coral larva (pyriform shaped) settling and, (**B**) photo of an adult colony, showcasing its potential for larval emission.

The study area includes the region between Cabo de São Tomé (21.9 ∘S) and Ilha Grande Bay (23.4 ∘ S) focusing on the coast of Rio de Janeiro (Fig 2). The main goal is to examine how larval dispersion occurs from coastal source points such as rocky shores and ports associated with offshore oil and gas activities, as well as the influence of submesoscale structures on their pathways. Some anthropogenic structures act as potential vectors for the dispersion of sun coral larvae when vessels transit between these exploitation facilities near the coast, particularly in ports. In Fig 2 are shown four potential coastal sites for sun coral larvae release: Arraial do Cabo (AC), Ilha Grande Bay (IGB), Açú Harbor (AH) and Cagarras Islands (CI). Açu Harbor was chosen as source point due to its operation by vessels from the oil and gas industry, and reports of the significant presence of sun coral (*Tubastraea* spp.) in both natural rocky environments and artificial substrates. Offshore oil fields in the Campos Basin, identified as potential larval arrival sites, are marked by green polygons. Two key points, P1 and P2, associated with these fields, were clustered for analysis (Fig 2).

The Regional Ocean Model System (ROMS) serves as the primary model employed in this study [33], used to simulate the hydrodynamic fields within the study area. ROMS is a three-dimensional regional model that employs the primitive equations of motion in terrain-following vertical coordinates. ROMS is a modular framework that accommodates the inclusion of atmospheric and tidal components, as well as integration with other global models at its open boundaries. Our implementation follows the progression established by [10]. Here, we used three nested grids, as outlined in Fig 2. The model is forced using 3-hour atmospheric forcing from ERA5 [34], daily oceanic forcing at the boundaries of GLORYS12V1 [35], and astronomical tide from the TPXO global tide model [36,37]. The experiment aims to be representative of local dynamics, so the tide is included. However, exploring the influence of the tide is not the primary focus of the study. Tidal processes are imposed through sea level elevation and currents within the model’s open boundary conditions. The tide components that were included are M2, S2, O1, K2, K1, N2, P1, and Q1 on the Gchild high resolution grid. Tidal amplitude and phase data are provided as input so that ROMS can compute sea surface height and barotropic velocity at each time step across the model grid. The model’s time step varies based on the grid and nesting levels, starting at 180s for the parent grid, 60s for the child grid, and finally 12s for the Gchild grid, where dispersion is calculated. There are 32 vertical layers in each grid, with the stretching parameters $\theta_{b}$ and $\theta_{s}$ set to 0.45 and 4.5, respectively, and the output frequency is every six hours.

The submesoscale phenomena present in the Cabo Frio region was resolved through the three level nesting: a parent grid at $1/12^{\circ }$, a child grid at $1/36^{\circ }$, and a Gchild grid at $1/180^{\circ }$ (Fig 2). This hierarchical approach allowed us to capture the intricate details of eddies and filaments with varying degrees of precision. Through this gradual increase in horizontal resolution, we reached a grid spacing of 600 m within the Rio de Janeiro region (Gchild domain). This progressive refinement offers unparalleled insights into the submesoscale features prevalent in the region. Smaller scale phenomena, such as surface gravity waves, Langmuir cells and turbulence are not resolved by this model. This grid was specifically designed to resolve submesoscale phenomena which is the focus of our study. To identify submesoscale features, we look for regions with 𝒪(1) Rossby number (ζ/*f*), and normalized strain rate (α/|f|), where

$$\alpha ={\left[{\left(\frac{\partial u}{\partial x}-\frac{\partial v}{\partial y}\right)}^{2}+{\left(\frac{\partial v}{\partial x}+\frac{\partial u}{\partial y}\right)}^{2}\right]}^{1/2},$$ (1)

*u* is the zonal velocity, and *v* is the meridional velocity.

The validation of the hydrodynamic model is detailed in [10] by using real-world observational data, such as ARGO float measurements (S1 Fig) for the Gchild grid. The strong agreement between observed and simulated temperature-salinity structures highlights the model’s ability to accurately replicate the complex hydrography of the Brazil Current system, reinforcing its potential for broader ecological and environmental applications.

### Larvae dispersal model

We simulate the larvae dispersal with Lagrangian virtual particles using the ROMS module called FLOATS, which runs in parallel with the model (operating online) and tridimensionally. Additionally, a “random walk” subroutine is implemented to simulate the vertical motion of particles, accounting for unresolved turbulent transport. Sun coral larvae have reduced swimming abilities and feed on energy reserves stored in internal vesicles [38,39]. Due to this reduced swimming ability, the sun coral larvae are simulated as passive tracers, being transported by the model’s three-dimensional velocity field, thus allowing for variation in depth. The larvae are released in layer 32 (water’s surface) of the $1/180^{\circ }$ grid and their dispersion is computed at each ROMS time step. The number of particles (larvae) seeded at each point was 5,000, with 1,000 particles released each day for 5 days, following the study by [15].

We simulate two study cases with the objective of understanding the role of submesoscale dynamics on larvae dispersal. Since the time-scales of submesoscale processes can range from days to weeks, we choose a 20 day interval for each simulation. It has been shown, for several species of corals, that the larval competency period appears to be the primary factor influencing their spatial distribution [38]. The competency window is the lapse of time during which the larvae can settle on a substrate and successfully undergo metamorphosis into early juvenile recruits [40]. As these larvae do not feed on plankton, this period is constrained by the nutritional reserves allocated inside internal vesicles during embryogenesis [41]. Several studies have linked the larval period of competence window to the dispersal potential of organisms that emit non-planktonic larvae [42–44]. Sun coral larvae can spend up to 90 days alive in the water column [14,45], although a 20-day period is associated with a minimum probability of mortality for these larvae [46]. Thus, we consider this period appropriate to simulate the effect of submesoscale features on larvae dispersal, since most of the released larvae are expected to reach arrival sites alive. Our results show that the 20-day period is enough time for submesoscale currents to affect inter-connectivity of different location and to promote offshore export to oil and gas exploration regions along Rio de Janeiro’s coast.

As the implementation of the hydrodynamical model carries dated atmospheric and boundary conditions (ERA5, GLORYS12V01, and TPXO), simulation periods were also selected according to the spawning months reported for this genus in the study region, preferably summer months at full and new moons [47], although *Tubastraea* spawning are reported year round [14]. The results we obtained from the simulations are not exclusive to sun coral, but they apply to this organism.

### Experiments

Two experiments were conducted to assess dispersal from four potential source areas of sun coral larvae and areas suitable for colonization, based on the coastal hydrodynamics. We consider scenarios involving different wind conditions, which modulate the progression of coastal currents and submesoscale filaments originated in theses scenarios. The prevailing wind patterns in the region are the primary drivers of these currents, as explained by [11], and [48]. Thus, scenarios with different wind regimes are simulated to investigate how sun coral population in AC, AH, CI and IGB (Fig 2) are interconnected among each other and with offshore oil platforms.

**Fig 3:**
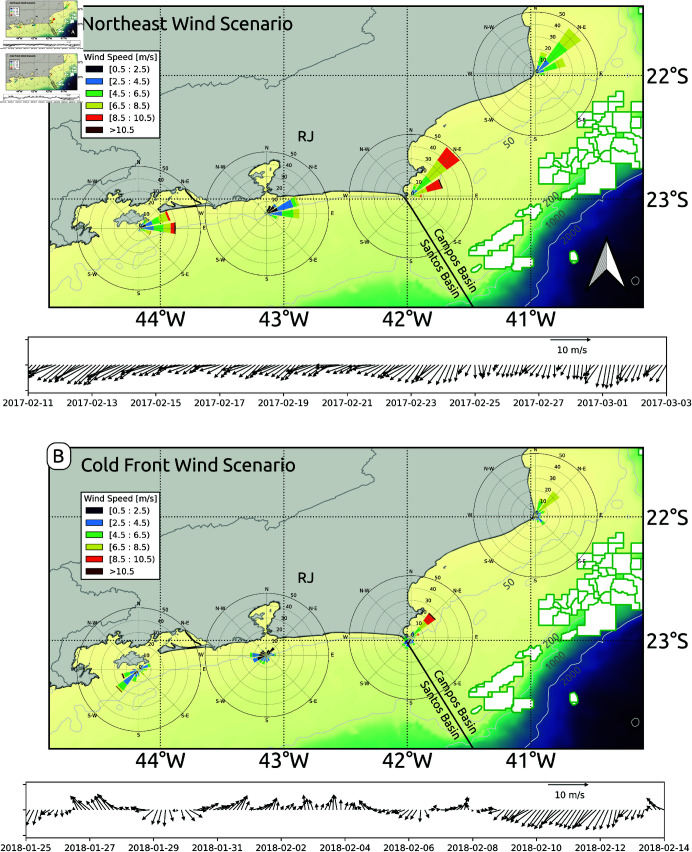
Wind patterns for two scenarios: (a) Northeast wind scenario and (b) Transition from northeast to cold front winds.

The frequent change in wind conditions in this region is due to the passage of atmospheric cold fronts that disturb the prevalent northeasterly winds, associated to the semipermanent South Atlantic High, to intermittent southwesterly winds. We designed our study case experiments to capture these two scenarios, dividing them into: (1) Prevailing Northeasterly Wind Scenario; and (2) Cold Front (Southwesterly) Winds Scenario (Fig 3). Both experiments were carried out for 20 days, focusing on how the fast submesoscale processes can modulate the initial dispersal of sun coral larvae. The dispersal period in the northeast scenario is from Feb 11 to Mar 3, 2017. The southwest scenario is from Jan 25 to Feb 14, 2018.

Fig 3 presents the wind roses at four potential key dispersal points of the invasive sun coral. Each panel corresponds to a specific scenario, displaying an average stick plot of the wind field over the high-resolution grid for the designated simulation periods. These stick plots provide a detailed view of the wind direction and speed, allowing for a comparative analysis of the meteorological conditions that shape larval dispersal patterns in the study area.

Fig 3a and 3b show distinct differences between the two proposed wind scenarios on the model simulations. Fig 3a captures the dominant northeast wind pattern, which is typical of the southeastern coast of Brazil. This northeast wind regime is associated with stable atmospheric conditions and a steady influence on surface currents. The consistent northeast wind plays a significant role in shaping nearshore hydrodynamics, fostering upwelling events, and influencing the local ocean circulation. This dynamics is crucial for understanding how *Tubastraea* spp. larvae are transported along the coast, since this wind-driven circulation creates opportunities for the larvae to remain in coastal waters or be exported offshore, contributing to their spread.

In contrast, Fig 3b illustrates a shift in wind patterns due to the arrival of a cold front, a common meteorological phenomenon in the region. As the cold front arrives, the northeasterly winds turn to the southwest, significantly altering the regional wind regime. This shift is critical for larval dispersal because it can disrupt the established northeast wind-driven currents and lead to a temporary reversal in surface flows. As the southwest cold front winds increase in intensity, they can generate new circulation features, such as downwelling near the coast or changes in the strength and direction of alongshore currents. The transition between northeast and cold front wind events observed in this region reflects the atmospheric variability, highlighting the complexity of coastal weather systems and their potential impact on larval transport.

To represent the connectivity between sources and arrival sites, we created the correlation matrix that was calculated based on predefined polygons of the locations in focus (circles in Fig 2), where the percentage of particles emitted and those that reach other areas are computed. Therefore, it is calculated in a unidirectional manner. It is important to note that particles are only emitted from the coastal sites, which will be presented in greater detail in the results section. Then, the connectivity calculations are based on the origin of the particles, so it is calculated in the coast-to-coast and coast-to-platform directions.

## Results and discussion

### Submesoscale regimes under different wind conditions

The presence of submesoscale features off Rio de Janeiro’s coast [31] holds the potential to facilitate the export of coastal larvae to offshore areas. Submesoscale motions can be triggered by flow-topography interactions [26,27] which, on the shelf, can be modulated by the wind-driven circulation that alters how currents interact with topography.

**Fig 4:**
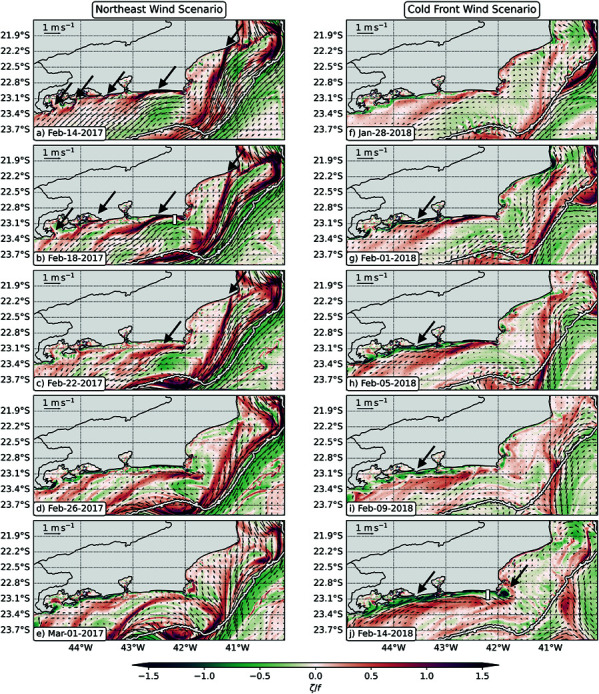
Submesoscale filaments and eddies off Rio de Janeiro’s coast for the Northeast wind (left column) and Cold Front wind (right column) scenarios. Horizontal maps of surface Rossby number (ζ/*f*) on: (**a**) Feb 14, 2017, (**b**) Feb 18, 2017, (**c**) Feb 22, 2017, (**d**) Feb 26, 2017, (**e**) Mar 1, 2017, (**f**) Jan 28, 2018, (**g**) Feb 1, 2018, (**h**) Feb 5, 2018, (**i**) Feb 9, 2018, and (**j**) Feb 14, 2018. The maps are constructed with daily averaged files. The white bars on panels (b) and (j) show the position of the vertical sections displayed on Fig 5. The white contours on all panels show the 200 m isobath, and the big dark arrows highlight the cyclonic (red) and anticyclonic (green) submesoscale filaments.

**Fig 5:**
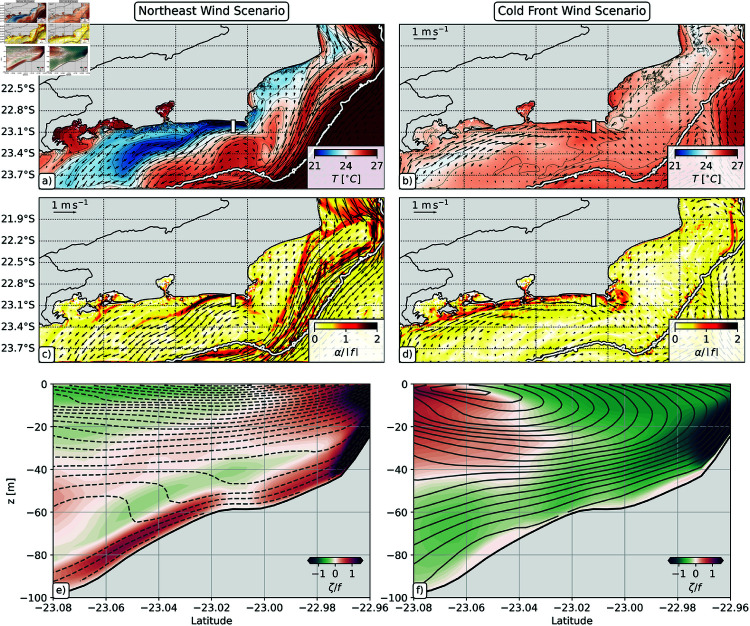
Submesoscale filaments for the NE wind case scenario on February 18, 2017 (left column) and for the Cold Front wind scenario on Feb 14 2018 (right column). The maps are constructed with daily averaged files. Horizontal maps of: (**a**– **b**) sea surface temperature, and (**c**– **d**) normalized strain rate (α/|f|). The white contours on panels a-d show the 200 m isobath. Vertical sections of: (**e**– **f**) Rossby number (ζ/*f*). The position of the vertical sections displayed on panels (e) and (f) is shown as white bars on panels (a)–(d) and on Fig 4b and 4j. The solid (dashed) isolines on panels (e) and (f) represent lines of constant eastward (westward) velocities.

Under northeast winds, the coastal circulation is characterized across the whole shelf by southwestward flowing currents. In this scenario, several filaments form and transport cold upwelling water southwestward (Figs 4a–4c and 5a). The most prominent filament originates at Cabo Frio (42 ∘ W), although other filaments are also observed along the coast, e.g., (i) Cabo de São Tomé (41 ∘W); (ii) west of Baía de Guanabara (43.5 ∘ W); and (iii) Ilha Grande Bay (44.1 ∘ W).

These filaments are generated on separation regions [23,49], where the coastal current interacts with topography increasing lateral shear and vorticity (Figs 4a–4c and 5e). Intense cyclonic vorticity is ejected from CF, giving rise to the submesoscale filaments (the narrow regions with 𝒪(1) ζ/f; Fig 4e), where Lagrangian material typically accumulates due to enhanced strain rates (Fig 4c). Around February 26, 2017, the northeast winds weakened on the west part of the domain leading to less southwestward currents (Fig 4d). Consequently, submesoscale cyclonic filaments no longer dominate the shelf circulation west of Cabo Frio (42 ∘W). In fact, by March 1, we observe that a mesoscale cyclone forms in the Cabo frio region. This mesoscale cyclone brings southeastward currents to Arraial do Cabo and is another potential driver of larvae dispersal.

In the Cold Front Wind Scenario, the simulation starts with weak northeast winds (Fig 3b) and we observe weak cyclonic filaments on the shelf (Fig 4a). Around February 1, 2018, a cold atmospheric front enters the domain and the winds shift from northeast to southwest winds. This wind shift leads to northeastward currents that form narrow anticyclonic submesoscale filaments that remain trapped near the coast (Fig 4g). From February 5 to February 8, the winds shift to northeast winds again (Fig 3b), and some southwestward cyclonic filaments are ejected into the offshore region (Fig 4h) from Cabo de São Tomé (41 ∘W) and Cabo Frio (42 ∘W).

This fluctuating pattern reveals that when Cold Atmospheric Fronts arrive in the region, the anticyclonic filaments might act to trap the larvae near the coast. This is confirmed by the stronger normalized strain rates near the coast (Fig 5d), that restrict the accumulation of larvae to the nearshore region. However, the shift to northeast winds might still allow some larvae to escape due to the formation of the southwestward cyclonic filaments (Fig 4j).

Eventually, if the Cold Front Winds are persistent, the northeastward currents can also eject submesoscale features offshore. In this scenario, the lateral shear against topography amplifies anticyclonic vorticity (Fig 4j and Fig 5f), and we observe the formation of submesoscale anticyclonic features downstream of Cabo Frio (Fig 5f, similarly to [50]). Even though this was not observed in our study case, these anticyclones could trap particles and travel offshore, also contributing to larvae export.

To understand how these submesoscale filaments and eddies affect the dispersion of sun coral larvae on this region, we analyze the trajectories of simulated Lagrangian particles in each experiment. We focus on the effect of horizontal transport, but we also acknowledge that the different wind scenarios and submesoscale regimes can also affect temperature (Fig 5a and 5b) and, thus, larvae metabolic rates might also be affected due to changes on environmental conditions. The results of our larvae dispersion experiments are shown on the next section.

### Larval dispersion Along Rio de Janeiro’s coast

The larvae dispersion experiments show that larvae transport is mainly driven by the submesoscale regimes modulated by the wind-driven circulation. We observe that different regimes affect patterns of offshore transport, near coast entrapment and, consequently, connectivity between different sites.

**Fig 6:**
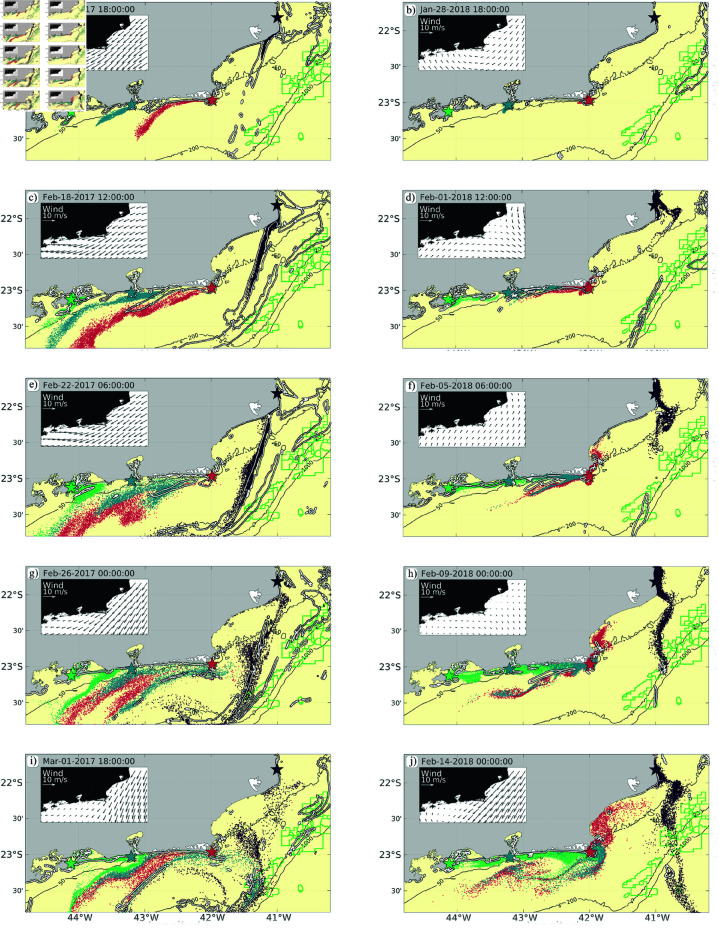
Larval dispersal simulation for each dispersal point in the wind direction scenario (northeast wind and cold front wind). The maps are constructed with instantaneous files. The colors of the derivatives indicate their region of origin. The white lines correspond to the Rossby number O(1).

Under Northeast wind conditions, the dispersion patterns along the shelf revealed that larvae tend to move southwestward, with significant concentration in narrow filaments (Fig 6a–6c). The concentration of larvae is often co-located with regions of 𝒪(1) Rossby number, confirming that submesoscale filaments play a major role on opening transport pathways for larvae dispersal across the shelf [30]. The concentration of material on filaments is a typical feature of submesoscale currents [28,29,12] due to the strong strain rates associated to these processes (Fig 5c).

**Fig 7:**
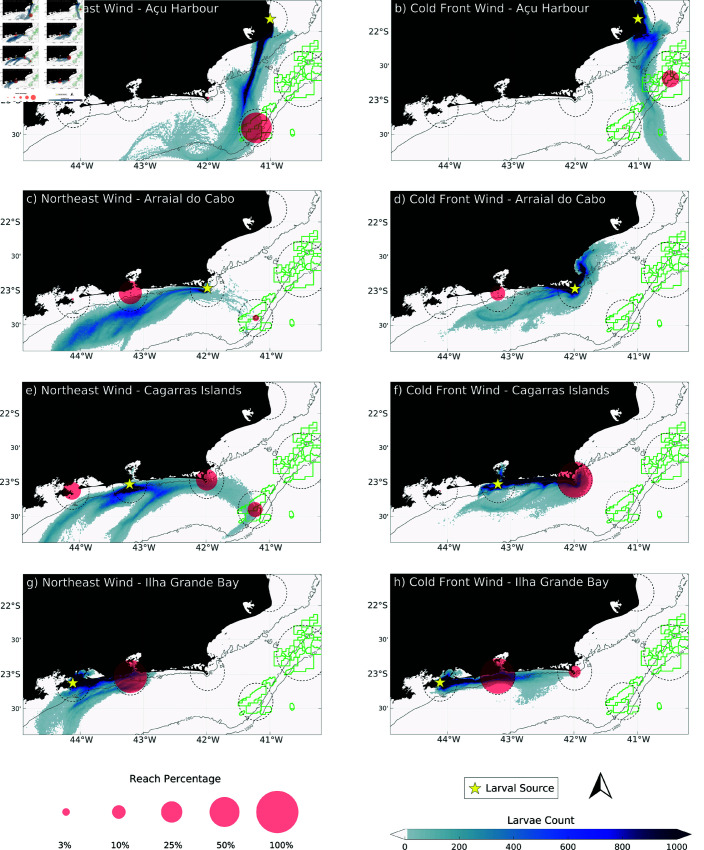
Integrated density plume of larval dispersion (blue plume) for each wind direction (Northeast wind and cold front wind). The pink circles indicate the final population of larvae at the end of the simulation.

This scenario favors the offshore transport of larvae besides supporting southwestward transport from AC to CI and from CI to IGB (Figs 6a, 6c, 6e and 7c, 7e). Here, we observe that larvae can be transported from the coast towards oil platforms as exemplified by the drifters from AH that reach P1 (Figs 6e, 6g and 7a). After the northeast winds weaken at the western portion of the domain, northeastward currents contribute to advect IGB larvae to CI (Figs 6i and 7g) and, moreover, the formation of a mesoscale cyclone off Cabo Frio supports larvae transport from CI to AC, and from AC to P1 (Fig 4e and 6i).

In the scenario characterized by a shift to Cold Front winds, a markedly different dispersal pattern emerges. The southwest wind regime tends to concentrate larvae closer to the coast and lead to northeastward transport (Fig 6b, 6d, 6f), altering the dispersal pathways observed under northeast winds. In this scenario, larvae originating from IGB, in Sepetiba region and the Cagarras Islands, near Guanabara Bay, exhibit a strong tendency to remain within the coastal zone (Figs 6d, 6f, 6h, 6j and 7h). This retention is likely driven by the reversal of surface currents, which now favor an onshore movement, trapping larvae in nearshore environments. However, this scenario is characterized by fluctuating conditions due to the alternation between cold front winds and the prevalent northeast winds. Thus, some larvae can still escape the near-coast entrapment and be transported offshore by cyclonic filaments (Figs 6f, 6h, 6j and 7d, 7f, 7h). Consequently, even in this scenario coastal larvae from AH can reach oil exploration regions (Figs 6h, 6j and 7b).

**Fig 8:**
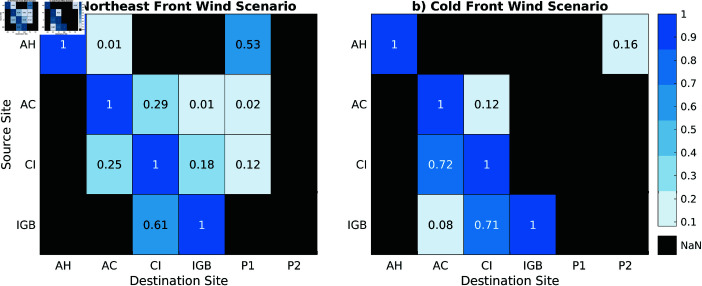
Connectivity matrix of larval dispersal.Results for: (a) Northeast Wind Scenario, and (b) Cold Front Wind Scenario.

Fig 8a and 8b presents connectivity matrices depicting the relationship between the source and destination sites for larval dispersal under two distinct wind scenarios. These matrices provide a quantitative analysis of the dispersion patterns and the concentration of larvae at various destination sites, reinforcing the earlier observations about how wind regimes influence larval transport. The percentage values in these matrices highlight the extent of connectivity between source regions and the final settlement areas for larvae.

In the northeast wind scenario, illustrated in Fig 8a, there is a higher spread of larvae, with the dispersion extending into offshore regions. Notably, there is connectivity between P1 and coastal sites like AH, AC, CI, with values of 0.53, 0.02 and 0.12, respectively. This indicates that the northeast winds, through their capacity to generate offshore-directed submesoscale filaments, are capable of transporting larvae to distant offshore locations. The connectivity values, while lower than those observed in coastal regions, confirm that larvae are reaching oil and gas exploration zones. This offshore transport, facilitated by submesoscale processes and wind-driven currents, underscores the potential for *Tubastraea* spp. larvae to establish populations on offshore infrastructure, thus expanding their range beyond the coastal zone.

In contrast, the cold front wind scenario, shown in Fig 8b, reveals a more constrained dispersal pattern, with larvae predominantly concentrated along the coast. The correlation values between source and destination sites reflect this limited dispersal range, particularly in the connections between the CI and the AC region (0.72), as well as between IGB and the CI (0.71). These percentage values exceeding 0.7 suggest that larvae are less likely to be transported offshore in this wind scenario, remaining confined to nearshore regions where coastal currents and local hydrodynamics dominate their movement.

The contrasting dispersal patterns observed in the two wind scenarios highlight the critical role of wind-driven circulation in shaping the submesoscale transport pathways of *Tubastraea* spp. larvae. Successful recruitment and further colonization are strongly related to temperature, salinity, light, substrate availability, and calcite concentration [51,52], so it goes beyond the implications of our findings. However, the presence of export filaments in the northeast wind scenario suggests that coastal development and offshore infrastructure are inherently connected through these dispersal mechanisms. Offshore oil and gas platforms, which serve as artificial substrates for *Tubastraea* spp., can act as a stepping stone for the spread of the species. This underscores the importance of monitoring larval transport pathways in both coastal and offshore environments, particularly in regions where human activities, such as oil and gas exploration, may inadvertently facilitate the expansion of invasive species. Additionally, [53] points out that anthropogenic structures such as port areas, shipyards, and marinas in Guanabara Bay facilitate the spread of non-native species.

In Brazilian waters, high larvae connectivity was found between offshore oil and gas basins that are subjected to the regional Brazil Current flow [9], however our results focus on the coastal dispersion affected mainly by the wind-driven currents and submesoscales filaments. Furthermore, the larvae dispersion of *Tubastraea coccinea* in their southern Atlantic limit of distribution was investigated by [54]. The authors used simulated currents to investigate the possible places in the expansion of this species in a Biological Marine Reserve. They found that none of the larvae released from oil platforms reached the coast, so they raise the discussion of colonization through the vessel flux between these offshore structures and the Itajaí harbor.

Although this study considered only two scenarios, they are representative of the prevailing wind-driven currents patterns in Rio de Janeiro’s coast in a real-world context. The animations in S2 (northeast scenario) and S3 (cold front scenario), which present simulations over a longer timeframe, reveal that larvae can reach areas beyond our established arrival sites. By incorporating more scenarios into future simulations, and resolving smaller scale dynamics not included in this model, different dispersion dynamics are likely to emerge, leading to an even broader range of possible trajectories. This underscores the complexity and variability of larval dispersal processes, which are influenced by multiple environmental and anthropogenic factors.

## Conclusion

This study provides an examination of the natural dispersion mechanisms of *Tubastraea* spp. larvae along the southeastern coast of Brazil, focusing on the significant role of wind-driven currents and submesoscale oceanic processes. Through the use of high-resolution numerical modeling, we demonstrate how coastal dynamics especially submesoscale features, such as filaments and eddies critically influence cross-shelf exchange. These filaments facilitate the transport of larvae from coastal areas to offshore regions, including oil platforms. This work highlights the complex mechanisms by which biological invasions, particularly involving marine species like *Tubastraea* spp., are facilitated by combined oceanographic and human factors. Our simulations apply not only to sun coral larvae, but also to other passive tracers and larvae that have a competency period of at least 20 days.

A key aspect of our findings is the influence of wind patterns on larval dispersion. Northeasterly winds, which are predominant in the region, drive southwestward submesoscale filaments that transport larvae offshore. These dynamic features enhance the probability of larvae being transported far from their original coastal habitats, increasing the potential for colonization of distant, offshore ecosystems, including artificial structures like oil platforms. In contrast, southwesterly winds, typically associated with the passage of cold fronts, tend to trap the larvae near the coast, transporting them to northeast. The interaction between wind-driven currents and submesoscale phenomena promotes connectivity between coastal sites that are historically invaded by sun coral, thus playing a significant role in shaping the patterns of *Tubastraea* spp. larval distribution. During cold front scenarios, the connectivity between the Cagarras Islands and Ilha Grande Bay sites are strong (0.70), showing that IGB serves as source of larvae to CI. In similar wind conditions, CI is also an important source of larvae to Arraial do Cabo (0.72 connectivity). The cross-shelf connections are established from Açú Harbor promoting coastal larvae supply to offshore regions related to oil and gas productions (0.52).

In addition to natural oceanographic processes, we draw attention to the influence of offshore oil and gas activities as vectors for the spread of invasive species. Model results reveal a clear connectivity between coastal *Tubastraea* spp. populations and offshore installations, suggesting that larvae may be transported either directly by ocean currents or indirectly through human activities, such as the movement of support vessels and industrial operations. The potential for invasive species to colonize offshore structures, such as oil rigs, is of particular concern, as these artificial habitats can facilitate the persistence and further spread of *Tubastraea* spp. populations in marine environments far from their point of origin. This underscores the importance of considering both natural and anthropogenic factors in the management of invasive species.

In this study, we opt to simulate high-resolution computationally-expensive scenarios that resolve submesoscale processes. In exchange, we are limited by the short duration of our simulations. To constrain the dispersal sensibility to specific scenarios, ensemble simulations [9,55] with different hydrodynamic scenarios, spawning dates, and testing different depth releases [56] would be of great value to build upon the findings described in these simulations. Furthermore, additional effort is necessary to: (i) improve the representation of larvae behavior in numerical models (e.g., including the effect of temperature on larvae mortality rates); and (ii) validate the dispersal mechanisms described here with observations.

Overall, this study enhances the understanding of how coastal oceanographic features influence the distribution of invasive species in marine environments. We elucidate the complex interplay between wind-driven currents, submesoscale features, and anthropogenic factors, and provide critical insights that can inform conservation efforts and the sustainable management of marine resources. Future research should continue to confirm the genetic connectivity among different locations [57], and to investigate the multifaceted interactions between natural and human-induced factors in biological invasions. The findings of this study are particularly relevant for policymakers, conservationists, and industries seeking to balance economic activities with the protection of marine biodiversity.

## Supporting information

Fig. S1 Model validation. Comparative T–S diagram showing data points from the ROMS model (black markers) and ARGO profilers (blue markers), covering the period from 2010 to 2019
(PDF)

Fig. S2 Animation of larval dispersal under cold front wind conditions (southwest wind). Simulation of larval dispersal from each release point under the cold front (southwest wind) scenario. The animation covers the period from Jan, 25, 2018 to 14, Fev, 2018. The colors of the trajectories indicate their region of origin. The white lines represent areas with a Rossby number of order O(1).
(Zip)

Fig. S3 Animation of larval dispersal under northeast wind conditions. Simulation of larval dispersal from each release point under the northeast wind scenario. The animation covers the period from Fev, 11, 2017 to 03, Mar, 2017. The colors of the trajectories indicate their region of origin. The white lines represent areas with a Rossby number of order O(1).
(Zip)
