# Correction: Simulated larvae dispersion of the invasive sun-coral (*Tubastrea* spp.) along Rio de Janeiro’s coast: The role of submesoscale filaments on offshore transport and connectivity

**DOI:** 10.1371/journal.pone.0332059

**Published:** 2025-09-10

**Authors:** Leandro Calado, Bernardo Cosenza, Francisco Moraes, Caique Dias Luko, Damián Mizrahi, Fabio C. Xavier, Daniela Batista, Roberto Domingos, Sávio Calazans, Fernanda Araújo, Ricardo Coutinho

The genus name “Tubastraea” is misspelled in the article title. The correct title is: Simulated larvae dispersion of the invasive sun-coral (*Tubastraea* spp.) along Rio de Janeiro’s coast: The role of submesoscale filaments on offshore transport and connectivity. The correct citation is: Calado L, Cosenza B, Moraes F, Dias Luko C, Mizrahi D, Xavier FC, et al. (2025) Simulated larvae dispersion of the invasive sun-coral (*Tubastraea* spp.) along Rio de Janeiro’s coast: The role of submesoscale filaments on offshore transport and connectivity. PLoS One 20(6): e0313240. https://doi.org/10.1371/journal.pone.0313240.
