# Supplementary material for: Simulated larvae dispersion of the invasive sun-coral (Tubastrea spp.) along Rio de Janeiro’s coast: The role of submesoscale filaments on offshore transport and connectivity
Source: PLoS One. 2025 Jun 5;20(6):e0313240. doi: 10.1371/journal.pone.0313240 (PMC12140225; doi:10.1371/journal.pone.0313240)
Supplement: Fig. S1 — Comparative T–S diagram showing data points from the ROMS model (black markers) and ARGO profilers (blue markers), covering the period from 2010 to 2019 (PDF) [file pone.0313240.s001.pdf]

Supplementary Material S1

1 MODEL VALIDATION

Data obtained from the ARGO profiler's Drifters project played a fundamental role in validating the model's implementation for the most refined grid. This validation process involved an extensive examination of temperature and salinity data extracted from in situ profiles of ARGO drifters within the latitude range of 22°S-28°S. Through this comparative analysis, we assessed the model's capacity to accurately replicate the stratification observed in the study area.

The analysis incorporated a set of 2010-2019 individual observations, compared against averages for each simulated period. This comparison, illustrated by the T-S diagram (Fig. S1), clearly demonstrates the alignment of the primary oceanic water masses, consistent with previous studies. The model generally exhibits a satisfactory representation of the thermohaline structure, reflecting its robustness in capturing the essential dynamics of the Brazil Current region.

Further discussion reveals that the ARGO data were crucial in pinpointing specific areas where the model's performance could be improved. For instance, slight discrepancies in the upper thermocline's temperature gradients suggested a need for finer resolution or enhanced parameterization of mixing processes. Additionally, the salinity profiles highlighted regions where freshwater input or evaporation effects might require more detailed representation.

Overall, the integration of ARGO profiler data significantly enhanced the credibility of our model simulations. The consistency between observed and simulated temperature-salinity structures underscores the model's capability in accurately reproducing the complex interactions within the Brazil Current system. This validation not only affirms the model's current performance but also provides a foundation for future refinements and applications in similar coastal and regional oceanographic studies.

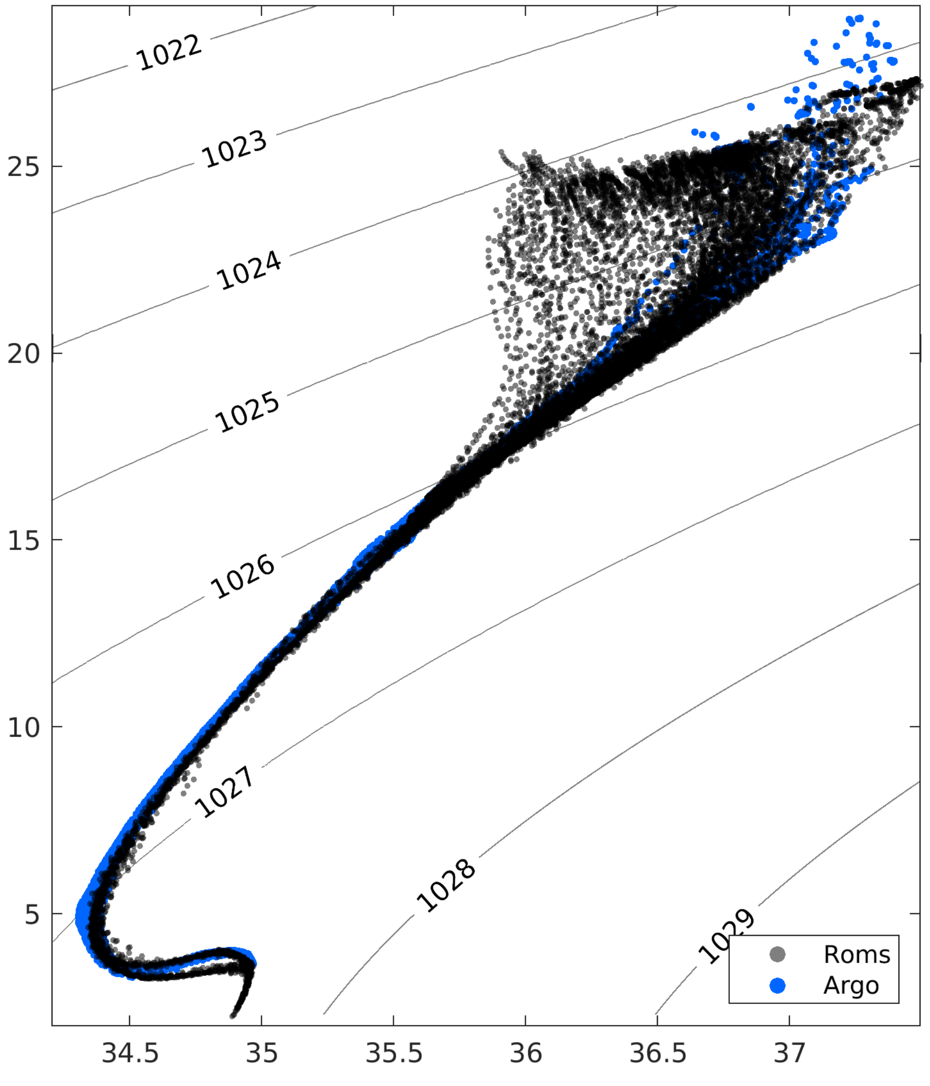

Figure S1. Comparative T-S diagram is presented, featuring data points from ROMS indicated by black markers and those from ARGO profilers by blue markers, spanning the years from 2010 to 2019
