# Supplementary figures and images for: Simulated larvae dispersion of the invasive sun-coral (Tubastrea spp.) along Rio de Janeiro’s coast: The role of submesoscale filaments on offshore transport and connectivity
Source: PLoS One. 2025 Jun 5;20(6):e0313240. doi: 10.1371/journal.pone.0313240 (PMC12140225; doi:10.1371/journal.pone.0313240)

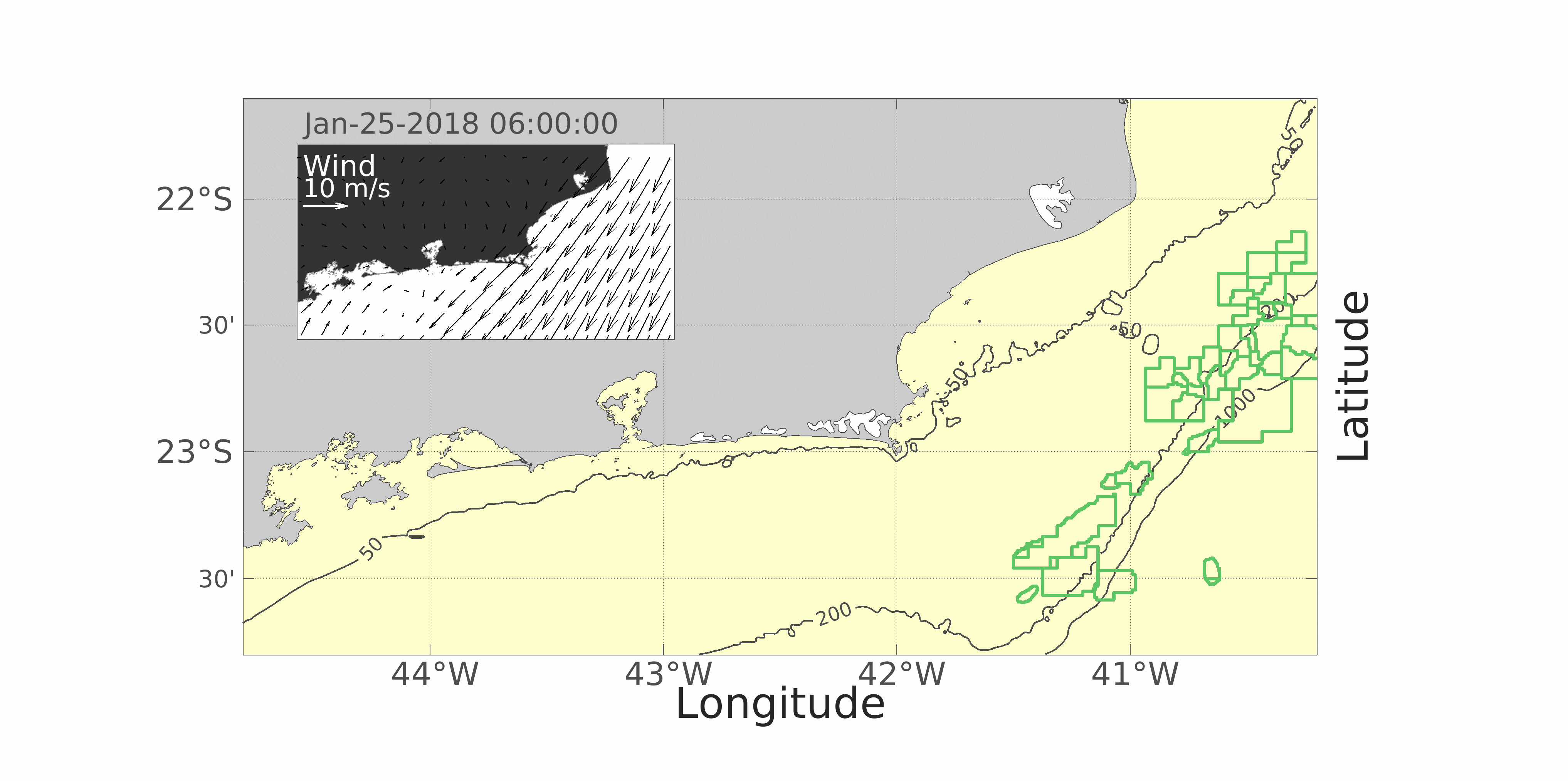

Supplement: Fig. S2 — Simulation of larval dispersal from each release point under the cold front (southwest wind) scenario. The animation covers the period from Jan, 25, 2018 to 14, Fev, 2018. The colors of the trajectories indicate their region of origin. The white lines represent areas with a Rossby number of order O(1). (Zip) [file pone.0313240.s002.zip › s2_animation_cold_front_wind.gif]

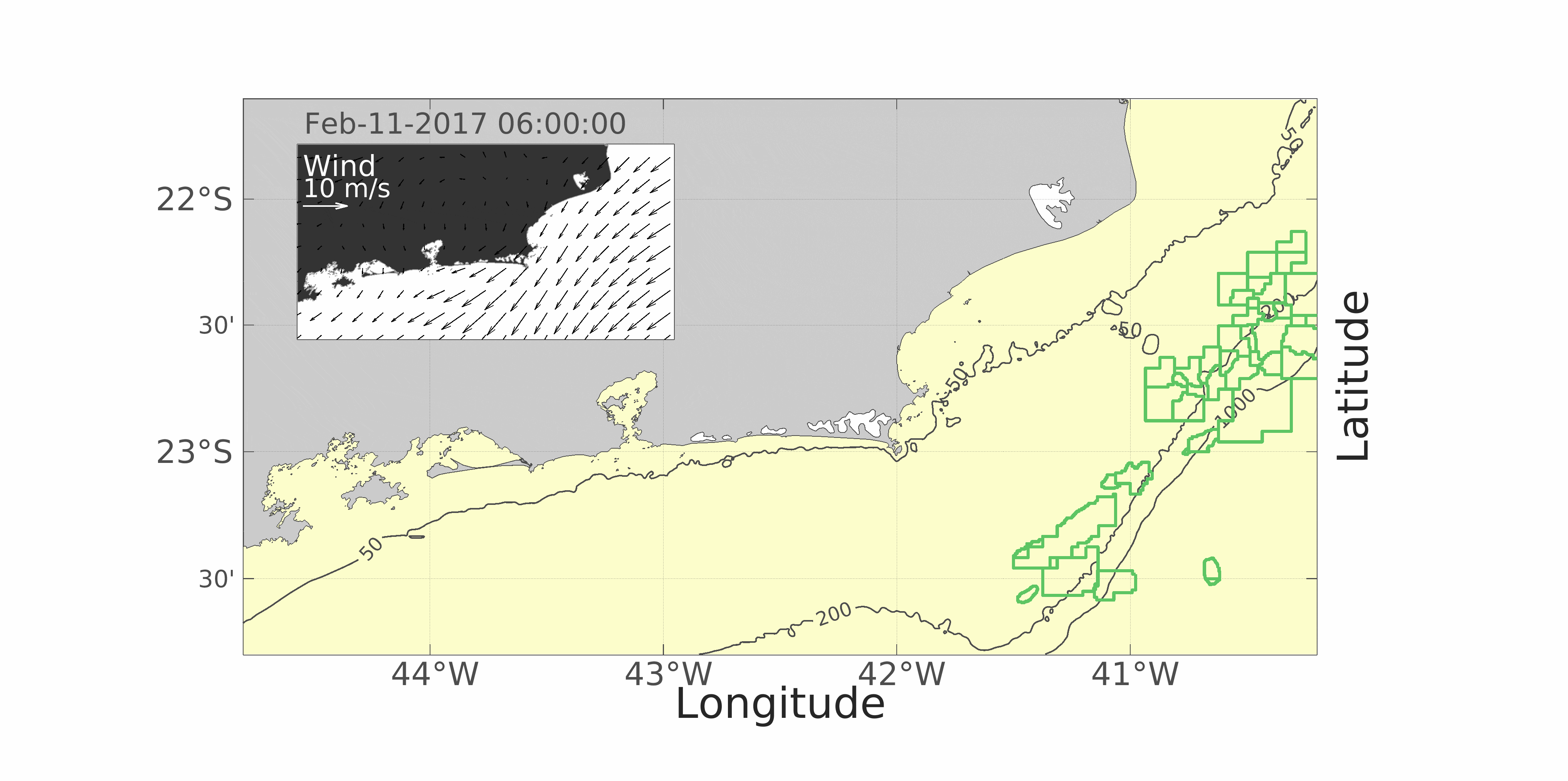

Supplement: Fig. S3 — Simulation of larval dispersal from each release point under the northeast wind scenario. The animation covers the period from Fev, 11, 2017 to 03, Mar, 2017. The colors of the trajectories indicate their region of origin. The white lines represent areas with a Rossby number of order O(1). (Zip) [file pone.0313240.s003.zip › S3_animation_ne_wind.gif]
